# Acute kidney injury, baseline renal function, troponin, and survival after myocardial infarction

**DOI:** 10.3389/fneph.2026.1761672

**Published:** 2026-08-19

**Authors:** Bing Lu, Jacqueline P. Honerlaw, Yuk-Lam Ho, Daniel Posner, David R. Gagnon, Xuan-Mai T. Nguyen, Jason L. Vassy, J. Michael Gaziano, Kelly Cho, Luc Djousse, Peter W. F. Wilson

**Affiliations:** 1 Massachusetts Veterans Epidemiology Research and Information Center (MAVERIC), VA Boston Healthcare System, Boston, MA, United States; 2 Department of Public Health Sciences, University of Connecticut Health Center, Farmington, CT, United States; 3 Department of Biostatistics, Boston University School of Public Health, Boston, MA, United States; 4 Department of Medicine, Brigham and Women’s Hospital, Harvard Medical School, Boston, MA, United States; 5 Department of Medicine, New England Geriatric Research Education and Clinical Center (GRECC), Boston, MA, United States; 6 Atlanta VA Medical Center, Atlanta, GA, United States; 7 Emory University Schools of Medicine and Public Health, Atlanta, GA, United States

**Keywords:** acute kidney injury, mortality, myocardial infarction, renal function, troponin

## Abstract

**Background:**

The roles of chronic kidney disease and acute kidney injury (AKI) as determinants of short- and long-term survival following myocardial infarction (MI) have not been well investigated.

**Methods:**

A retrospective analysis of electronic health record data was conducted for U.S. veterans between 2002 and 2015, with up to 15 years of follow-up after MI. Eligible participants included 49,150 veterans with a serum creatinine measurement prior to MI hospitalization. AKI stage was determined by the change between the pre-hospitalization creatinine level and the peak in-hospital measurement. The associations of AKI, estimated glomerular filtration rate (eGFR), and troponin I ratios with survival were evaluated using Cox proportional hazards models.

**Results:**

Compared to the no-AKI group, the hazard ratios (HRs) and 95% confidence intervals (CIs) for 30-day mortality were 3.12 (2.87–3.39), 5.12 (4.65–5.65), and 7.81 (6.93–8.81) across worsening stages of AKI. For 15-year mortality, the HRs were 1.76 (1.68–1.85), 2.33 (2.17–2.50), and 2.66 (2.37–2.99). Compared to those with eGFR >60 mL/min/1.73 m², HRs (95% CI) for 30-day mortality were 1.23 (1.13–1.33), 1.46 (1.33–1.59), and 1.92 (1.72–2.13) across decreasing eGFR levels. For 15-year mortality, HRs were 1.21 (1.17–1.25), 1.55 (1.49–1.61), and 2.13 (2.03–2.24). Mortality rates increased with rising troponin I (cTnI) ratios, particularly for 30-day mortality.

**Conclusions:**

AKI, baseline eGFR, and troponin levels were associated with both short- and long-term survival following MI. AKI during MI hospitalization was a particularly strong determinant of short-term mortality.

## Introduction

Refinement of prognostic algorithms to predict mortality risk following acute coronary syndrome (ACS) or myocardial infarction (MI) has led to the identification of baseline kidney function and acute kidney injury (AKI) as important determinants of survival. (1) Chronic kidney disease (CKD) that was present prior to an MI event and AKI during the hospitalization have been shown to affect both short- and long-term mortality risk. (2) Given the rising prevalence of CKD and the risk for AKI during inpatient hospitalizations (3, 4), these factors are now recognized as important determinants of post-MI outcomes.

The definitions of kidney function and AKI in the literature are inconsistent and vary based on the study period and the data available. A recent meta-analysis of AKI in ACS reported four AKI definitions in use (1). Definitions of CKD are typically based on serum creatinine measured at the time of the MI or ACS, which may inaccurately estimate renal function prior to the occurrence of the MI. Additionally, patients with CKD experience greater risk of AKI due to decreased kidney reserve (3, 4). It is important for clinicians to correctly interpret the prognostic value of estimated glomerular filtration rate (eGFR) and AKI on MI prognosis.

The U.S. veteran population is burdened by CKD and cardiovascular comorbidities at a higher rate than the general population, and the electronic health record (EHR) for veterans provides laboratory information concerning renal function prior to and during hospitalizations for MI (5). The primary aim of this analysis was to determine how pre-hospitalization baseline renal function and AKI contribute to post-MI mortality in a veteran population.

## Methods

### Study design

We performed a retrospective analysis on EHR data from the Veterans Health Administration (VHA), the largest integrated health system in the United States. VHA EHR data are stored in the central Corporate Data Warehouse, which contains healthcare data for veterans across the United States. This study was approved by the Institutional Review Board at the VA Boston Healthcare System.

The study population included veterans hospitalized in the VHA system between 2002 and 2015 with an inpatient discharge diagnosis of MI [International Classification of Diseases 9th Edition (ICD-9), code 410.x or 411.x] (6). Only first inpatient VA MI events were included in this analysis. We excluded veterans with cancer (ICD-9 codes 140.00–239.00, except non-melanoma skin cancers 173.2, 173.4, and 173.9) because they might be receiving treatments (e.g., chemotherapy and radiation) that also impact kidney function. Patients with a procedure code for blood vessel surgery, including coronary artery bypass surgery or percutaneous angiography with or without stent placement (ICD-9 procedure code 36.xx) 5 days before measurement of peak cardiac troponin I (cTnI), were excluded to ensure that increased troponin levels were not related to a potential post-surgical elevation in troponin. Additionally, we excluded dialysis patients and those with kidney failure at baseline (eGFR < 15 mL/min/1.73 m^2^). Veterans were included in the analysis if the duration of the MI hospitalization was less than 1 year and if the veteran had at least two of the same cardiac biomarkers (troponin I, troponin T, creatine kinase MB, or creatine kinase) measured during the MI hospitalization period. Study participants were required to have a serum creatinine measurement in the 2 years prior to their MI hospitalization and troponin I measurement collected during their MI hospitalization and at least one creatinine measured during the 3 days following the peak cTnI. The final analytic cohort consisted of 49,150 hospitalizations for MI.

### Exposures of interest

We calculated the eGFR using the equation developed by the Chronic Kidney Disease Epidemiology Collaboration (7). The serum creatinine used to calculate eGFR was identified from the closest inpatient or outpatient visit prior to the MI hospitalization, at a maximum of 2 years before admission. AKI during the MI hospitalization period was defined using a modified AKI staging scale based on Kidney Disease: Improving Global Outcomes (KDIGO) guidelines (8). Because this retrospective study relied on routinely collected EHR data, the exact timing required to apply the KDIGO absolute increase criterion (≥0.3 mg/dL within 48 h) was not consistently available, and urine output data were unavailable or incompletely recorded across VA hospitals. AKI classification was based on relative changes in serum creatinine only. Serum creatinine change was calculated by subtracting the serum creatinine obtained prior to hospitalization from the peak serum creatinine measurement within the 3-day interval following the day of the peak troponin ratio (cTnI-R). Categories of serum creatinine change were calculated with <50% for no AKI, 50%–99% for Stage 1 AKI, 100%–199% for Stage 2 AKI, and ≥200% for Stage 3 AKI. All laboratory measures were adjudicated by two clinician reviewers to harmonize results across VHA laboratories.

Serum cTnI assays have varying reference values. The ratio of the cTnI measurement to the reference upper limit of normal (cTnI-R) was calculated using assay-specific reference ranges available in the EHR to maximize the comparability of cTnI measurements across patients. The peak cTnI-R for the MI hospitalization period was selected from these calculated ratios, yielding one peak cTnI-R value per admission. Given the time period used in this analysis, high-sensitivity cTnI measures were not available for this cohort. The peak cTn-R measurement served as a proxy for the date of the MI during the hospitalization.

### Mortality and comorbidities

All-cause mortality after peak cTnI-R was ascertained from the National Death Index (NDI) through the end of 2016 (9). Comorbid conditions and medications were defined using EHR data. The presence of at least one ICD-9 code was used to indicate diabetes (250.x) and mental health conditions including posttraumatic stress disorder, schizophrenia, and bipolar disorder (309.81, 295, 301.2, 313.2, 296.0, 296.4, 296.5, 296.6, 296.7, 296.8, and 331.0). Systolic blood pressure and heart rate were averaged over outpatient VA clinic encounters within a year prior to hospitalization for MI.

### Statistical analysis

Baseline characteristics were described using frequencies, means, standard deviations, medians, and interquartile ranges stratified by eGFR and AKI stage. Data from veterans with the healthiest eGFR and AKI categories (≥60 mL/min/1.73 m² and no AKI) were compared to patients with the most compromised kidney function (15 to <30 mL/min/1.73 m² and AKI Stage 3) for a comparison of baseline health.

Short-term survival was calculated using person time of follow-up from the peak cTnI-R date through death or censoring date of 30 days. The long-term survival model followed veterans until death or administrative censoring based on NDI data through December 2016. Kaplan–Meier (KM) curves for long-term survival were stratified by eGFR categories (15–29, 30–44, 45–59, and 60+ mL/min/1.73 m²), AKI stage, and quartiles of peak cTnI-R (Q1: <1.25; Q2: 1.25–7.33; Q3; 7.33–49.0; and Q4: >49.0).

Cox proportional hazard models were used to predict short-term and long-term mortality across categories of peak cTnI-R, baseline eGFR, and AKI stage. No AKI, eGFR ≥60 mL/min/1.73 m², and the first cTnI-R quartile served as the reference group. To adjust for correlations between patients treated at the same hospital, we used a robust sandwich estimator. We performed crude and age-adjusted analyses, then applied multivariable adjustment for calendar year, age, length of hospital stay, region, diabetes, major mental health conditions, systolic blood pressure, heart rate and baseline use of diuretics, anti-hypertensives, anti-anginal therapy, and lipid medications. The long-term survival analysis included all participants, and a sub-analysis excluded veterans who died during the first-year after MI. Additionally, we created combined groups based on AKI, eGFR, and cTnI-R categories. Separate models were used to evaluate potential synergistic associations. To assess possible effect modification, we conducted stratified analyses for each exposure variable by age and race groups. Analyses were performed using SAS software (version 9.4; SAS Institute).

## Results

Among 49,150 hospitalizations for MI, 98.7% were male, 18.4% were Black, the average age was 67 years, and the median follow-up interval through the censoring date of 31 December 2016 was 4.4 years (interquartile range: 1.6 to 8.1 years). There were 4,231 deaths within 30 days of the peak cTnI-R measurement and 27,840 deaths during the entire follow-up interval. Older age, longer hospitalization, a higher proportion of Black veterans, and the presence of diabetes or diuretic use at baseline were more common in veterans with reduced eGFR or later AKI stages at MI hospitalization. Other clinical factors, such as mental health conditions, heart rate, anti-anginal use, and lipid medications, also had differential distributions across eGFR categories and AKI stages (**Table 1**).

**Table 1:** Baseline characteristics of study sample according to renal function and acute kidney injury*.

Baseline characteristics		Acute kidney injury (AKI) stage**				Estimated glomerular filtration rate (eGFR) mL/min/1.73 m²			
		No AKI	Stage 1	Stage 2	Stage 3	60+	45-59	30-44	15-29
		*n* = 43,016	*n* = 3,571	*n* = 1,685	*n* = 878	*n* = 31,321	*n* = 9,119	*n* = 5,878	*n* = 2,832
Age at admission, years		66.7 ± 11.6	70.3 ± 11.8	70.9 ± 11.9	68.5 ± 11.9	63.5 ± 10.6	72.1 ± 10.7	75.1 ± 10.7	74.1 ± 11.3
Sex, male %		98.2	98.0	97.6	98.0	98.4	98.1	97.5	97.8
Hispanic, %		6.2	7.0	7.6	7.1	6.4	5.3	6.1	8.0
Race, %	Black	18.2	18.4	19.6	19.8	19.8	16.0	14.7	18.3
	White	80.6	79.9	78.7	72.9	79.2	82.5	83.9	79.2
	Other	1.2	1.8	1.7	0.6	1.1	1.5	1.5	2.5
Region, %	Southeast	20.5	19.4	21.0	22.4	20.8	19.8	19.8	20.3
	North Atlantic	21.5	21.3	22.3	20.6	21.0	22.4	22.8	22.1
	Midwest	21.0	21.1	21.3	18.0	20.5	21.1	22.5	22.6
	Continental	21.9	22.6	22.2	25.5	22.9	21.3	19.7	19.8
	Pacific	15.1	15.6	13.3	13.4	14.8	15.4	15.2	15.1
BMI, kg/m²		29.1 ± 6.5	28.6 ± 7.2	28.1 ± 7.4	28.5 ± 7.9	29.2 ± 6.7	28.9 ± 6.4	28.4 ± 6.5	28.0 ± 6.5
Length of hospitalization, days		6.8 ± 26.2	13.3 ± 44.8	13.4 ± 27.0	16.3 ± 26.5	6.9 ± 26.5	8.4 ± 36.5	9.1 ± 16.5	11.0 ± 33.1
eGFR, mL/min/1.73 m²		68.9 ± 23.0	64.9 ± 25.9	66.5 ± 26.0	77.3 ± 32.9	83.0 ± 15.1	53.0 ± 4.3	38.2 ± 4.2	23.5 ± 4.3
HR, beats/min		74.9 ± 11.9	75.9 ± 11.8	76.9 ± 12.1	77.4 ± 12.0	76.0 ± 12.0	73.8 ± 11.7	72.9 ± 11.4	72.7 ± 10.8
SBP, mmHg		135.7 ± 16.2	137.1 ± 17.0	136.5 ± 16.9	136.9 ± 16.5	135.3 ± 15.8	135.8 ± 16.3	137.1 ± 17.1	140.5 ± 17.9
DBP, mmHg		75.3 ± 10.3	73.1 ± 10.2	72.8 ± 9.9	74.0 ± 10.3	76.7 ± 10.1.	72.9 ± 9.9	71.2 ± 9.7	71.4 ± 10.2
Anti-hypertensive use, %		95.3	95.9	94.8	93.5	93.9	97.2	97.8	98.8
Major mental health condition, %		18.7	19.4	20.1	21.1	21.1	16,3	13.6	13.3
Anti-anginal use, %		58.9	55.4	49.3	42.3	55.4	61,2	63.1	67.0
Lipid medication use, %		83.1	79.0	75.4	66.2	81.6	83.0	84.0	83.8
Diabetes, %		48.2	59.1	57.6	51.0	44.9	52.7	59.5	66.9
Diuretic use, %		59.5	72.4	73.1	70.5	52.7	69.6	79.3	88.1

*Values are mean ± SD, %. AKI, acute kidney injury; BMI, body mass index; eGFR, estimated glomerular filtration rate; HR, heart rate; SD, standard deviation; SBP, systolic blood pressure; DBP, diastolic blood pressure; VHA, Veterans Health Administration.

**No AKI: serum creatinine change <50%; Stage 1: 50%–99%; Stage 2: 100%–199%; Stage 3: ≥200%.

**Figure 1:**
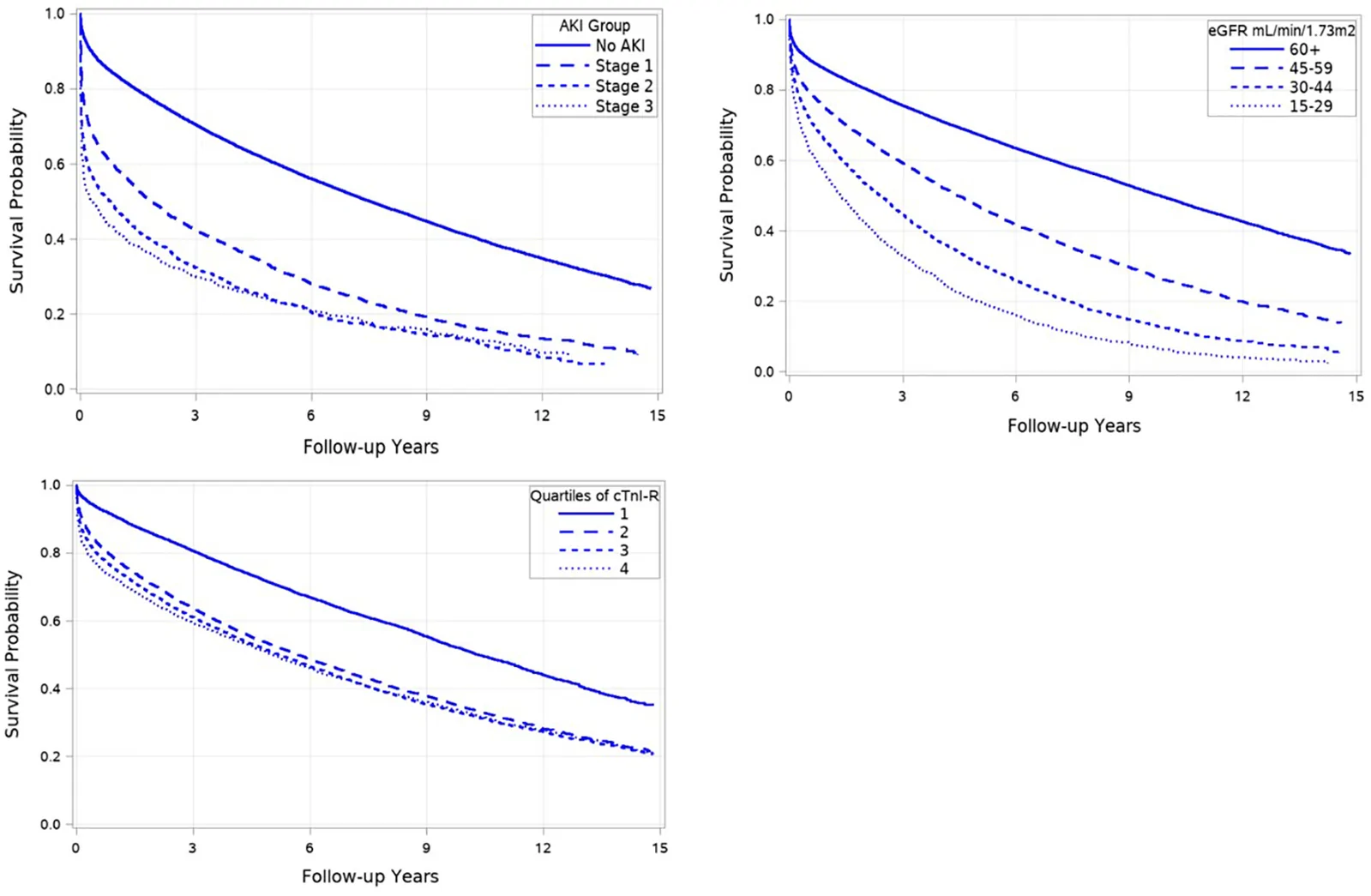
Survival probability through 2016 stratified by AKI stages, eGFR groups and quartiles of peak troponin I ratio.

**Figure 1** shows the KM survival curves through 2016, stratified by AKI stages, eGFR groups, and quartiles of peak troponin I ratio. A rapid decline in survival shortly after MI was observed among veterans with AKI, and the effects worsened in proportion to AKI severity, highlighting its significant association with mortality. The survival curves stratified by eGFR categories showed distinct trajectories, with worse survival at lower eGFR levels. Patients with eGFR ≥60 mL/min/1.73 m² exhibited the highest survival probability. Similarly, the KM curves stratified by peak cTnI-R quartiles showed significantly lower survival probabilities for quartiles 2–4 compared to the first quartile. Survival rates of patients in peak cTnI-R quartiles 2, 3, and 4 converged after 3 years.

The overall associations of AKI stage, eGFR category, and peak cTnI-R quartile with short- and long-term mortality were analyzed with multivariable Cox proportional hazards models, and survival was reduced in proportion to worse AKI stage, eGFR category, and peak cTnI-R quartile (**Table 2**). In the short-term 0- to 30-day survival analysis, the magnitude of effect was greatest for AKI stage compared to eGFR and cTnI-R. Multivariable adjusted HRs (95% CI) were 1.0 (reference—No AKI), 3.12 (2.87, 3.39), 5.12 (4.65, 5.65), and 7.81 (6.93, 8.81) across worsening stages of AKI. In the long-term survival analysis, AKI again had the largest magnitude of effect [multivariable adjusted HRs (95% CI): 1.0 (ref), 1.76 (1.68, 1.85), 2.33 (2.17, 2.50), and 2.66 (2.37, 2.99)]. However, in a long-term survival sub-analysis that excluded veterans who died in the first year of follow-up, eGFR had the strongest association on mortality compared to AKI stage and peak cTnI-R quartile: multivariable adjusted HRs (95% CI) were 1.0 (reference), 1.22 (1.17, 1.27), 1.63 (1.55, 1.71), and 2.23 (2.08, 2.40) across increasing categories of eGFR. Similarly, mortality risk was higher among a higher level of cTnI-R, demonstrating a clear dose–response relationship. In the short-term (0 to 30 days) survival analysis, the magnitude of association was greater compared to that observed in the long-term survival analysis. Multivariable-adjusted HRs with 95% CIs for short-term survival were 1.00, 2.00 (1.76–2.27), 2.56 (2.26–2.90), and 3.34 (2.95–3.78) across increasing levels of the cTnI ratio. In contrast, the HRs (95% CI) for long-term survival were 1.00, 1.38 (1.34–1.43), 1.46 (1.41–1.51), and 1.50 (1.45–1.56).

**Table 2:** Hazard ratios (HR) and 95% confidence intervals (CI) of short- and long-term mortality after MI*.

Groups		Short-term (0–30 days)		Long-term (0–15 years)		Long-term (Year 1–Year 15)	
		4,231 deaths/49,150 veterans		27,840 deaths/49,150 veterans		17,574 deaths/38,984 veterans	
		HR**	95% CI	HR**	95% CI	HR**	95% CI
AKI**	No AKI	1.00	Reference	1.00	Reference	1.00	Reference
	Stage 1	3.12	2.87, 3.39	1.76	1.68, 1.85	1.44	1.35, 1.53
	Stage 2	5.12	4.65, 5.65	2.33	2.17, 2.50	1.61	1.46, 1.77
	Stage 3	7.81	6.93, 8.81	2.66	2.37, 2.99	1.40	1.19, 1.66
eGFR** (mL/min/1.73 m²)	60+	1.00	Reference	1.00	Reference	1.00	Reference
	45-59	1.23	1.13, 1.33	1.21	1.17, 1.25	1.22	1.17, 1.27
	30-44	1.46	1.33, 1.59	1.55	1.49, 1.61	1.63	1.55, 1.71
	15-29	1.92	1.72, 2.13	2.13	2.03, 2.24	2.23	2.08, 2.40
Quartiles of peak cTnI-R**	Q1: <1.25	1.00	Reference	1.00	Reference	1.00	Reference
	Q2: 1.25–7.32	2.00	1.76, 2.27	1.38	1.34, 1.43	1.29	1.24, 1.35
	Q3: 7.33–49.0	2.56	2.26, 2.90	1.46	1.41, 1.51	1.31	1.25, 1.37
	Q4: >49.0	3.34	2.95, 3.78	1.50	1.45, 1.56	1.26	1.20, 1.32

*MI, myocardial infarction; AKI, acute kidney injury; eGFR, estimated glomerular filtration rate; cTnI-R, cardiac troponin I ratio. No AKI: serum creatinine change <50%; Stage 1: 50%–99%; Stage 2: 100%–199%; Stage 3: ≥200%.

**Adjusted for calendar year, age, length of hospitalization, geographic region, diabetes, mental health conditions, systolic blood pressure, heart rate, diuretics, anti-hypertensives, anti-anginals, and lipid medications.

**Supplementary Table 1** shows the associations of the combined groups based on AKI, eGFR, and cTnI-R categories with long-term mortality. Compared to veterans with no AKI and eGFR ≥60 mL/min/1.73 m², a higher stage of AKI combined with a lower eGFR was significantly associated with a much greater mortality risk. For example, the group with Stage 3 AKI and an eGFR of 15 to <30 mL/min/1.73 m² had the highest HR (95% CI) of 5.01 (3.50, 7.16). Higher stage of AKI combined with a higher cTnI-R was also significantly associated with a much higher mortality risk. Compared to veterans with no AKI and in the first quartile of cTnI-R, those in Stage 3 AKI and the highest quartile of cTnI-R had an HR (95% CI) of 4.05 (3.32, 4.93). In stratified analyses by age and race (**Supplementary Table 2**), the associations of AKI, eGFR, and cTnI-R with long-term mortality were consistent with the overall analyses above. Specifically, the magnitude of the AKI or eGFR association with long-term mortality was greater in veterans < 65 years old compared to those ≥65 years. However, no significant differences were observed between White and Black racial groups.

## Discussion

Our results show that both AKI that reflects acute changes in renal function and eGFR measured prior to an MI are important determinants of short-term and long-term mortality after MI in veterans. The mortality risk increases with worsening renal function and is statistically significant after adjustment for troponin quartile, demographic factors, comorbidities, and medication use. AKI and higher troponin levels were strongly associated with short-term mortality, while lower baseline eGFR assessed in an outpatient setting prior to an MI was strongly associated with long-term mortality.

The different associations of AKI and baseline kidney function may reflect distinct underlying pathophysiologic processes. AKI occurring during MI hospitalization likely reflects the severity of the acute clinical insult, including hemodynamic instability, reduced renal perfusion, systemic inflammation, and other complications associated with acute MI. In contrast, lower baseline eGFR measured before hospitalization reflects underlying chronic kidney dysfunction and the cumulative burden of cardiovascular disease and other comorbidities. Among patients who survive the acute phase of MI, these chronic conditions may play a greater role in determining long-term prognosis, which may explain why baseline eGFR showed a stronger association with long-term mortality.

These findings build on previous reports concerning the impact of pre-existing renal function AKI on post-MI mortality. First, AKI is relatively common at the time of an MI, affecting 13% of veterans in this report and approximately 16% of MI or ACS victims, as described in published articles and in a meta-analysis that included more than 100,000 MI victims (1, 10, 11). Other investigations have assessed the role of baseline kidney function and prognosis after MI. A study compared different definitions of AKI in patients with acute MI and suggested that AKI was associated with long-term mortality, regardless of the definition used (12). In a single-center retrospective cohort study based on data from 1,655 consecutive patients diagnosed with acute MI, AKI was strongly associated with both short- and long-term all-cause mortality, regardless of baseline renal impairment. Notably, a dose–response relationship between AKI severity and short-term mortality was observed (13).

The ACTION registry investigators, analyzing Medicare claims data in the 2008–2012 interval, reported that AKI was associated with 1-year mortality in a cohort of 76,000 inpatient hospitalizations in persons >65 years and that CKD at baseline modified this effect (2). Those authors used the KDIGO definition to define AKI during hospitalization and the Modification of Disease in Renal Disease (MDRD) eGFR equation to define CKD. We used a similar approach to assess AKI. The ACTION registry used the serum creatinine collected at the time of MI admission to define eGFR and excluded MI victims on dialysis at baseline. We used the Chronic Kidney Disease Epidemiology Collaboration equation to estimate eGFR, which adjusts for body surface area as well as race, age, and gender to estimate baseline kidney function (14).

The ACTION authors reported a significant interaction between AKI and eGFR in their cohort, which had follow up from hospital discharge up to 1 year after MI. We also identified similar interactions for the associations of both AKI and eGFR with survival, which was evident for both long-term (*p* < 0.001 for interaction) and short-term (*p* < 0.001 for interaction) survival. The ACTION study population was composed of a Medicare eligible population, whereas 51% of our cohort was >65 years old. Our long-term subgroup analysis excluded deaths in the first year, and the magnitude of the eGFR association with mortality was greater in veterans < 65 years old. We also observed a greater adverse relationship for AKI on survival in veterans < 65 years old in the long-term survival analysis. Our cohort was also more racially diverse (19% other than White compared to 8% in the ACTION cohort), and a stratified analysis did not show a difference in the risk between Black and White veterans. Another key difference in our study participants was that the ACTION cohort included records from 2008 to 2012 while ours had an expanded study interval with more recent data (2002–2015) and our long-term analyses include survival beyond 1 year after MI. The mortality rates across our cohorts are not directly comparable given that the ACTION registry focused on 1-year mortality and required survival through the end of hospital discharge, while our follow-up began at the time of the peak cTnI-R measurement.

We identified an interaction between the associations of cTnI and eGFR with survival (results not shown) and the biological explanation is not clear. A similar interaction between troponin T and renal function has been previously described in a study of post-operative mortality after non-cardiac surgery, but the selective nature of pre-surgical screening and the use of troponin T assay limit the comparability to our study (15). Miller-Hodges and colleagues tested, but did not identify, an interaction between baseline eGFR and cTnI (16). The conflicting evidence on the mechanism for troponin elevation in patients with impaired kidney function further complicates a biologic rationale for this interaction—clearance of troponin may be influenced by low kidney function or elevated troponin may be attributable to underlying heart disease in patients with low GFR that is underdiagnosed or subclinical (17–21).

Using VHA EHR data for a retrospective cohort study offers significant advantages, particularly due to its large sample size and longitudinal design (22). The VHA is the largest integrated healthcare system in the United States, providing access to rich clinical records, including diagnoses, medications, lab results, and imaging reports, in a cost-efficient manner (23) Additionally, VHA records are linked to the NDI and CMS to enhance the accuracy of mortality and healthcare utilization outcomes.

There are several limitations of our analysis. First, the population under study is predominantly older and male, and has a higher burden of comorbidities, limiting generalizability of our results to non-veteran populations. We are not able to make firm conclusions concerning the role of kidney function and post-MI survival for women due to small sample size (data not shown). Second, AKI definition did not incorporate the absolute serum creatinine increase (≥0.3 mg/dL within 48 h) or urine output criteria. The study may potentially under-identify Stage 1 AKI. Such nondifferential misclassification would likely bias associations toward the null. Third, our study is observational and susceptible to unobserved confounders. Although we adjusted for peak troponin level, length of hospitalization, blood pressure, heart rate, diabetes, and other clinical covariates, detailed measures of MI severity were not consistently available for analysis. However, adjustment for peak cTnI-R, which reflects infarct size and myocardial injury, likely captured an important component of disease severity. Also, we cannot determine whether observed associations are causally responsible for the increased mortality. These mechanisms nevertheless provide biologically plausible explanations for the strong association between AKI and early mortality observed in our study. Fourth, MI was defined using an ICD-9-based definition and may be susceptible to misclassification. Use of billing codes to define MI also prevented us from discerning between ST-elevation MI and non-ST-elevation MI. Additionally, we used the date of the peak cTnI-R measurement rather than the exact onset of MI as the temporal reference point. Some temporal imprecision in defining the index event is possible. However, because the primary objective was to evaluate prognostic associations rather than characterize the exact timing of MI, and because the same reference point was applied uniformly to all participants, any resulting bias is expected to be nondifferential and unlikely to materially affect the observed associations. Fifth, baseline kidney function was defined using the pre-hospitalization serum creatinine measured closest to the time of admission for MI and could reflect an acute change in the renal status of the patient.

In summary, our results support the use of AKI and eGFR staging with cTnI to predict mortality risk following MI (24). AKI is a stronger predictor than eGFR, particularly for short-term survival. Our large, nationwide sample enabled the study of a substantial number of patients diagnosed with MI compared to other studies that mainly used cohorts of patients presenting with ACS. The ability to assess the association of both kidney function measured before MI and acute change in kidney function during MI hospitalization with subsequent mortality is a unique aspect of our study, enabled by our access to serum creatinine measures from the EHR before and at the time of the MI event. We were also able to leverage data from a nationwide sample by using the ratio of the peak cTnI to the upper limit of normal of each assay to create a ratio that is comparable across VHA sites. Another strength of this analysis was the relatively high number of events observed in this cohort, which enabled high statistical power and robust association estimates.

## Conclusions

Baseline kidney function, AKI, and troponin levels are associated with short- and long-term survival after MI. Our findings suggest that acute changes in kidney function during the MI hospitalization and cardiac troponin level are strong predictors for short-term mortality, and baseline kidney function before the MI event is a determinant of long-term survival.

## Data Availability

The data analyzed in this study is subject to the following licenses/restrictions: VA investigators. Requests to access these datasets should be directed to vincisupport@va.gov.

## Supplementary material

Supplementary Table 1 Hazard Ratios (HR) and 95% Confidence intervals (CI) of Long-Term Mortality After MI According to eGFR Categories and Quartiles of Cardiac Troponin Ratio Stratified by Acute Kidney Injury*. * MI: Myocardial infarction; AKI: Acute Kidney Injury; eGFR: Estimated glomerular filtration rate; cTnI-R: Cardiac troponin I ratio. No AKI: serum creatinine change <50%; Stage 1: 50-99%; Stage 2: 100-199%; Stage 3: ≥200%. **Adjusted for calendar year, age, length of hospitalization, geographic region, diabetes, mental health conditions, systolic blood pressure, heart rate, diuretics, anti-hypertensives, anti-anginals and lipid medications.

Supplementary Table 2 Hazard Ratios (HR) and 95% Confidence intervals (CI) of Long-Term Mortality after MI by Age and Race*. *MI: Myocardial infarction; AKI: Acute Kidney Injury; eGFR: Estimated glomerular filtration rate; cTnI-R: Cardiac troponin I ratio. No AKI: serum creatinine change <50%; Stage 1: 50-99%; Stage 2: 100-199%; Stage 3: ≥200%. **Adjusted for calendar year, age, length of hospitalization, geographic region, diabetes, mental health conditions, systolic blood pressure, heart rate, diuretics, anti-hypertensives, anti-anginals and lipid medications.
