# Supplementary material for: Acute kidney injury, baseline renal function, troponin, and survival after myocardial infarction
Source: Front Nephrol. 2026 Aug 19;6:1761672. doi: 10.3389/fneph.2026.1761672 (PMC13533685; doi:10.3389/fneph.2026.1761672)
Supplement: Supplementary file 1 [file Table1.docx]

| **Supplemental Table 1. Hazard Ratios (HR) and 95% Confidence intervals (CI) of Long-Term Mortality After MI According to eGFR Categories and Quartiles of Cardiac Troponin Ratio Stratified by Acute Kidney Injury*** | | | | | | |  |
| --- | --- | --- | --- | --- | --- | --- | --- |
| **AKI** | **eGFR** | **HR**** | **95% CI** | **cTnI-R** | **HR**** | **95% CI** | |
| **No AKI** | 60+ | 1.00 | Reference | 1 | 1.00 | Reference | |
|  | 45-59 | 1.23 | 1.19,1.27 | 2 | 1.40 | 1.35, 1.46 | |
|  | 30-44 | 1.58 | 1.52,1.64 | 3 | 1.46 | 1.41, 1.52 | |
|  | 15-30 | 2.22 | 2.11,1.35 | 4 | 1.47 | 1.41, 1.53 | |
| **Stage 1** | 60+ | 1.84 | 1.73,1.96 | 1 | 1.73 | 1.51, 1.98 | |
|  | 45-59 | 2.13 | 1.92,2.36 | 2 | 2.25 | 2.06, 2.47 | |
|  | 30-44 | 2.76 | 2.47,3.08 | 3 | 2.63 | 2.43, 2.85 | |
|  | 15-30 | 3.42 | 2.98,3.93 | 4 | 2.77 | 2.53, 3.03 | |
| **Stage 2** | 60+ | 2.43 | 2.22,2.67 | 1 | 2.45 | 1.99, 3.03 | |
|  | 45-59 | 2.85 | 2.42,3.39 | 2 | 2.81 | 2.42, 3.26 | |
|  | 30-44 | 3.59 | 3.03,4.26 | 3 | 3.26 | 2.88, 3.69 | |
|  | 15-30 | 4.35 | 3.41,5,54 | 4 | 3.94 | 3.48, 4.45 | |
| **Stage 3** | 60+ | 2.99 | 2.59,3.45 | 1 | 2.78 | 1.96, 3.94 | |
|  | 45-59 | 2.71 | 2.05,3.58 | 2 | 3.88 | 3.14, 4.80 | |
|  | 30-44 | 3.45 | 2.47,4.80 | 3 | 3.68 | 2.99, 4.53 | |
|  | 15-30 | 5.01 | 3.50,7.16 | 4 | 4.05 | 3.32, 4.93 | |
| * MI: Myocardial infarction; AKI: Acute Kidney Injury; eGFR: Estimated glomerular filtration rate; cTnI-R: Cardiac troponin I ratio. No AKI: serum creatinine change <50%; Stage 1: 50-99%; Stage 2: 100-199%; Stage 3: ≥200%  **Adjusted for calendar year, age, length of hospitalization, geographic region, diabetes, mental health conditions, systolic blood pressure, heart rate, diuretics, anti-hypertensives, anti-anginal therapy and lipid medications. | | | | | | |  |

| **Supplemental Table 2. Hazard Ratios (HR) and 95% Confidence intervals (CI) of Long-Term Mortality after MI by Age and Race*** | | | | | | | | | |
| --- | --- | --- | --- | --- | --- | --- | --- | --- | --- |
|  |  | **Age ≥ 65 yrs**  18,331 deaths / 25,042 Veterans | | **Age < 65 yrs**  9,509 deaths / 24,108 Veterans | | **White**  22,609 deaths / 39,028 Veterans | | **Black**  4,627 deaths / 8,909 Veterans | |
|  |  | **HR**** | **95% CI** | **HR**** | **95% CI** | **HR**** | **95% CI** | **HR**** | **95% CI** |
| **AKI** | No AKI | 1.00 | reference | 1.00 | reference | 1.00 | reference | 1.00 | reference |
|  | Stage 1 | 1.73 | 1.65, 1.81 | 1.77 | 1.65, 1.91 | 1.78 | 1.69, 1.88 | 1.73 | 1.55, 1.93 |
|  | Stage 2 | 2.30 | 2.16, 2.46 | 2.20 | 1.99, 2.44 | 2.37 | 2.18, 2.57 | 2.12 | 1.81, 2.49 |
|  | Stage 3 | 2.43 | 2.11, 2.67 | 3.10 | 2.73, 3.52 | 2.75 | 2.38, 3.16 | 2.54 | 2.05, 3.15 |
| **eGFR (**ml/min/1.73 m²) | 60+ | 1.00 | reference | 1.00 | Reference | 1.00 | Reference | 1.00 | Reference |
|  | 45-59 | 1.11 | 1.07, 1.15 | 1.39 | 1.31, 1.48 | 1.18 | 1.14, 1.23 | 1.29 | 1.19, 1.40 |
|  | 30-44 | 1.38 | 1.33, 1.44 | 2.19 | 2.03, 2.37 | 1.53 | 1.47, 1.59 | 1.66 | 1.50, 1.84 |
|  | 15-30 | 1.91 | 1.81, 2.01 | 2.76 | 2.52, 3.02 | 2.16 | 2.04, 2.29 | 2.09 | 1.85, 2.37 |
| **Quartiles of Peak**  **cTnI-R** | Q1: <1.25 | 1.00 | reference | 1.00 | reference | 1.00 | reference | 1.00 | reference |
|  | Q2: 1.25-7.33 | 1.39 | 1.33, 1.46 | 1.38 | 1.30, 1.46 | 1.38 | 1.32, 1.43 | 1.44 | 1.32, 1.56 |
|  | Q3: 7.33-49.0 | 1.50 | 1.44, 1.57 | 1.40 | 1.32, 1.48 | 1.45 | 1.39, 1.50 | 1.52 | 1.39, 1.66 |
|  | Q4: >49.0 | 1.56 | 1.49, 1.63 | 1.41 | 1.33, 1.50 | 1.51 | 1.44, 1.57 | 1.49 | 1.36, 1.63 |
| *MI: Myocardial infarction; AKI: Acute Kidney Injury; eGFR: Estimated glomerular filtration rate; cTnI-R: Cardiac troponin I ratio. No AKI: serum creatinine change <50%; Stage 1: 50-99%; Stage 2: 100-199%; Stage 3: ≥200%  **Adjusted for calendar year, age, length of hospitalization, geographic region, diabetes, mental health conditions, systolic blood pressure, heart rate, diuretics, anti-hypertensives, anti-anginal therapy and lipid medications | | | | | | | | | |
